# Piroplasms in farmed American bison, *Bison bison* from Romania

**DOI:** 10.3389/fvets.2023.1158072

**Published:** 2023-03-29

**Authors:** Alexandra Corduneanu, Marian Taulescu, Teodor Dan Ursache, Angela Monica Ionică, Andrei Daniel Mihalca

**Affiliations:** ^1^Department of Animal Breeding and Animal Productions, University of Agricultural Sciences and Veterinary Medicine, Cluj-Napoca, Romania; ^2^Department of Pathology, University of Agricultural Sciences and Veterinary Medicine, Cluj-Napoca, Romania; ^3^Synevovet, Bucharest, Romania; ^4^Molecular Diagnosis Laboratory, Clinical Hospital of Infectious Diseases of Cluj-Napoca, Cluj-Napoca, Romania; ^5^Department of Parasitology and Parasitic Diseases, University of Agricultural Sciences and Veterinary Medicine, Cluj-Napoca, Romania; ^6^Parasitology Consultancy Group, Coruşu, Romania

**Keywords:** bison (North American bison), *Babesia*, *Theileria*, piroplasm, farmed

## Abstract

The American bison (*Bison bison*) is the largest terrestrial mammal of North America, with around 350,000 individuals in the wild and in private herds but the knowledge regarding the presence of different vector-borne pathigens in these mammals is very poor. *Babesia* and *Theileria* spp. are tick-borne apicomplexan parasites which are considered to be among the most commonly found blood parasites of large ruminants, often with a high economic importance. However, the knowledge on piroplasms of bisons is extremely scarce. The aim of our study was to evaluate the presence of apicomplexan parasites in blood and tissues of farmed American bison from Romania. Overall, we tested 222 blood samples and 11 tissues samples (heart, liver, and spleen) from farmed *B. bison* raised for meat in Romania. All the samples were analyzed by nPCR targeting the *18SrRNA* gene for piroplasmids. All positive samples were sequenced and analyzed phylogenetically. The overall prevalence of infection with piroplasmids in American bison was 1.65%, with *Babesia divergens* and *Theileria* sp. identified following sequencing. To our knowledge, this is the first report of piroplasms detected in blood and tissues of farmed *B. bison* from Europe. Further studies are necessary in order to obtain a better overview on the epidemiological status and clinical relevance of piroplasms in farmed American bisons.

## 1. Introduction

Bisons are large ungulates which became almost extinct by the beginning of the 20th century due to extreme hunting, habitat reduction and diseases transmitted from domestic livestock, especially cattle (1). They are the largest terrestrial mammals in North America and Europe. Currently there are two extant species: the American bison, *Bison bison* and the European bison or wisent, *Bison bonasus*, and six extinct species (*Bison palaeosinensis, Bison priscus, Bison schoetensacki, Bison antiquus, Bison latifrons*, and *Bison occidentalis*) which were present in south and north Asia, Europe, and North America (2–5). The number of bisons (*Bison bison*) from Romania is estimated to be around 1,500, all being raised for the meat industry.

The American bison is raised for meat production in a wide range of environments, using different management systems. The heard size ranges between 10 and several hundred individuals. These bisons are considered semi-domesticated, and their meat is highly appreciated due to the lack of antibiotics and hormones, low fat (<3%) and low cholesterol content (6).

Most studies regarding the diseases of American bisons were focused on viral and bacterial diseases, such as malignant catarrhal fever, brucellosis (particularly in the Yellowstone Park), anthrax, clostridiosis and vibriosis (7–9). In farmed bisons, the studies are even more scarce (10, 11). Surprisingly, there is only one field study reporting the presence of piroplasms in American bisons (12), followed by an experimental study (13).

Generally speaking, the most common piroplasms infecting large domestic ruminants (cattle, water buffalo) in North America are *Babesia bovis, Babesia bigemina* (14), and *Theileria orientalis* (15). Conversely, in Europe, the piroplasms more commonly reported in large ruminants are *Babesia divergens, Babesia major, Babesia occultans*, and *T. orientalis* (16). To the current date, there are no reports of piroplasmid infections in European bisons, nor in farmed American bisons from Europe. Hence, the aim of the present study was to evaluate the presence of piroplasmids in samples collected from *Bison bison* from Romania and to evaluate their phylogenetic relations with other piroplasms isolated from different large ungulates.

## 2. Material and methods

### 2.1. Sampling and DNA isolation

#### 2.1.1. Dead bisons

Between 2010 and 2015, a total of 36 dead adult bisons were brought to the Department of Pathology (Faculty of Veterinary Medicine Cluj-Napoca, Romania) for post-mortem examination. All individuals originated from a single location (Taga, Cluj County), where they were raised for meat. From the examined dead bisons, 13 cases were diagnosed (by PCR and microbiological tests) with various infectious diseases, including bovine viral diarrhea (BVD; *n* = 8), pulmonary pasteurelllosis (*n* = 3), and intestinal clostridiosis (*n* = 2). Cachexia related to severe infestation with hepatic flukes (*Fasciola hepatica*) and intestinal helminths (*Trichuris* spp.) was identified in seven cases. Three bisons died because of traumatic reticulopericarditis and peritonitis with intralesional metallic foreign bodies (Supplementary Figure 1). Fatal fight-related injuries were identified in other three individuals. In four bisons, because of advanced postmortem autolytic changes, the cause of death could not be established. The examination of the body surface of dead animals revealed the presence of hard ticks (Ixodidae) in three cases, which were collected and further morphologically identified using Olympus Bx51 microscope following the keys by Estrada-Pena et al. (17). Tissue samples were collected from six individuals with a suspicion of *Babesia* spp. infection, based on pathological findings. The collected tissue samples were represented by heart (*n* = 4), spleen (*n* = 3) and liver (*n* = 4). Unfortunately, in some animals, certain organs presented advanced stages of postmortem autolysis. Tissue samples were collected from individuals with a suspicion of *Babesia* spp. infection, based on gross pathological findings (e.g., splenomegaly, pale liver). Kidneys and lungs were collected for histological examination. On the other hand, in some animals, certain organs presented advanced stages of postmortem autolysis (especially the brain). Samples were first taken from dead bison and later, when lesions and signs of babesiosis were detected, blood samples were also taken from live animals to test for the presence of piroplasms. The clinical signs cannot be assessed because the animals roam freely in a very large area and cannot be cached. Samples were taken from dead bisons brought to necropsy by the owners and from live animals during blood collection for the national survey on diseases. DNA was extracted and tested for the presence of piroplasmids from each tissue sample. For cytology, multiple imprint smears from the spleen (*n* = 3) were stained with Dia-Quick Panoptic (Reagent Ltd., Budapest, Hungary) and subsequently examined using a high magnification objective (40 ×) and oil immersion (100 ×) to evaluate the morphological changes and to determine the presence of parasitic organisms. For histology, the samples were fixed in 10% phosphate buffered formalin for 24 h, embedded in paraffin wax, cut into 3 μm sections, and stained with hematoxylin and eosin (HE). Samples were examined using an Olympus Bx51 microscope and the photomicrographs were taken using an Olympus SP 350 digital camera and Stream basic imaging software (Olympus Corporation, Tokyo, Japan).

#### 2.1.2. Live bisons

**Figure 1:**
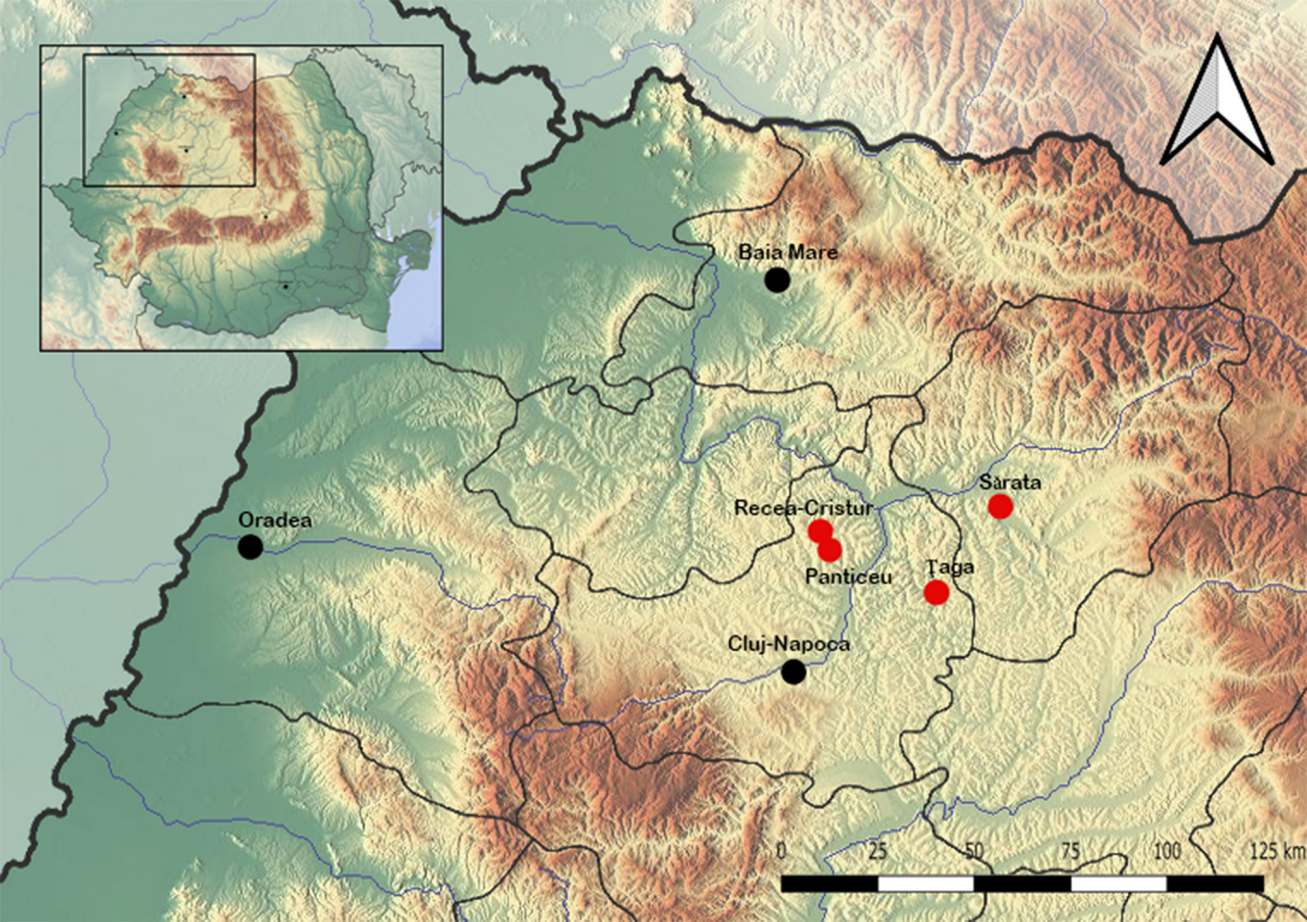
Collection sites.

Additionally, in order to evaluate the occurrence of piroplasmids in live bisons, a total of 222 blood samples were collected from American bisons between 2016 and 2017, originating from three farms, closely located to the farm from which dead individuals were obtained, where animals were also raised for meat (Figure 1). The blood was collected as part of the national strategic program for monitoring brucellosis and leucosis. The number of samples collected from each location was as follows: from Panticeu 66 samples, Recea Cristur 97 samples, and Sărata 59 samples. These numbers of samples collected represent the total number of animals that were on the farm at the time of collection. The bison are free-ranging and contact with ectoparasites, especially ticks, is very common. Collecting ticks from live animals is not possible because their size and temperament. For blood collection from live animals, they were held for a short period and not anesthetized.

### 2.2. DNA extraction

All blood samples were stored at −20°C until DNA extraction and the tissues in formalin for histopathological examination. Genomic DNA was extracted as soon as possible from 25 mg of heart or liver tissue, 10 mg of spleen tissue or 200 μl of blood, using Isolate II Genomic DNA Kit (Meridian Bioscience, London, United Kingdom) according to the manufacturer's instructions and stored at −20°C until further testing. The sample quantity for DNA extraction was established following the manufacturer's instructions and literature overview.

### 2.3. PCR amplification and electrophoresis

A nested PCR (nPCR) targeting a 561 bp fragment of the *18S rDNA* nuclear gene of piroplasmids was employed. The primers used for the nPCR were the following: BTH-1 F (5′-CCT GAG AAA CGG CTA CCA CAT CT-3′), and BTH-1R (5′-TTG CGA CCA TAC TCC CCC CA-3′) for the first round and GF2 (5′-GTC TTG TAA TTG GAA TGA TGG-3′), GR2 (5′-CCA AAG ACT TTG ATT TCT CTC-3′) for the second round. For PCR amplification the T1000™ Thermal Cycler (Bio-Rad, London, UK) was used with the following conditions: initial denaturation at 95°C for 3 min, then 40 cycles of denaturation at 95°C for 30 s, annealing at 60°C for 30 s (for the first round), 50°C for 30 s (for the second round) and extension at 72°C for 1 min (for the first round), 72°C for 40 s (for the second round) and a final extension at 72°C for 7 min (18). During the protocol, two negative controls (PCR water) were used for checking the possible contamination and one positive control consisting in DNA isolated from the blood of a naturally infected dog with *Babesia canis* (confirmed by sequencing) were included in each amplification set.

Amplification products were visualized by electrophoresis on 1.5% agarose gel stained with RedSafe™ 20,000 × Nucleic Acid Staining Solution (Chembio, UK), and their molecular weight was assessed by comparison to a molecular marker (HyperLadder^TM^ 100 bp, Bioline, London, United Kingdom).

### 2.4. DNA sequencing and phylogenetics analysis

Positive samples were purified using Gel/PCR DNA fragments extraction kit (Geneaid, Taipei, Taiwan) and sent for sequencing (performed by Macrogen Europe, Amsterdam, Netherlands). The sequences were compared with those available in GenBank™ by means of Basic Local Alignments Tool (BLASTn). All sequences were analyzed and edited using MEGA X software (19). The phylogenetic analysis was performed using the same software, using the Maximum Likelihood method: Tamura 3-parameter model (20), with a discrete Gamma distribution was used to model evolutionary rate differences among sites [five categories (+G, parameter = 0.2843)] for *Babesia* spp., and Tamura-Nei model (20), with a discrete Gamma distribution was used to model evolutionary rate differences among sites [five categories (+G, parameter = 0.2777)] for *Theileria* spp. The rate variation model allowed for some sites to be evolutionarily invariable [(+I), 39.05% sites].

For the phylogenetic analysis, all the sequences were trimmed to a length of 485 and 548 base pairs for *Babesia* and *Theileria*, respectively. In total, in the analysis we included 25 unique *Babesia* and 23 *Theileria* sequences from domestic and wild ungulates, including our sequences. *Hepatozoon* sp. was chosen as an outgroup.

## 3. Results

### 3.1. Pathological findings

**Figure 2:**
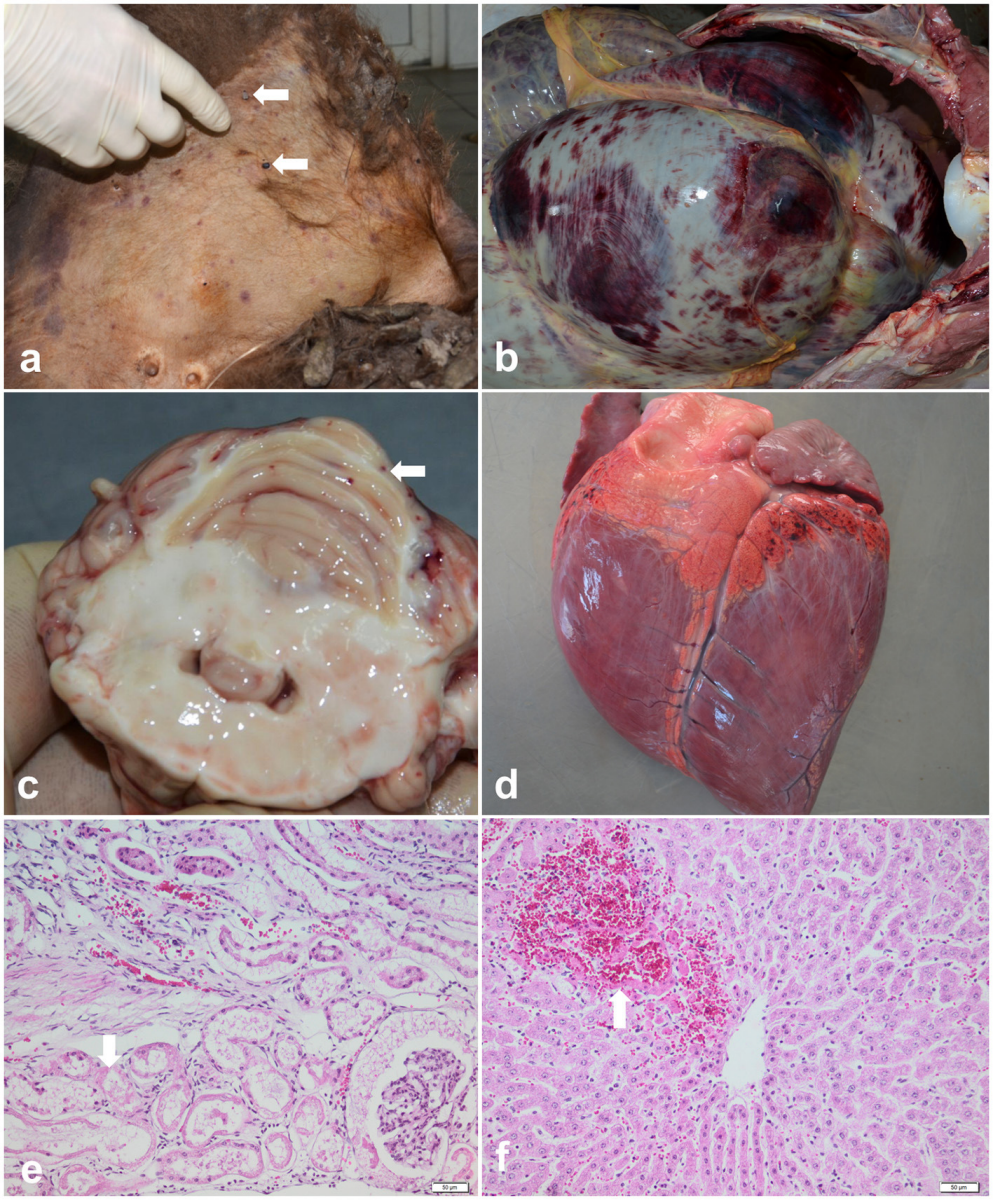
Pathological findings of babesiosis in American bisons (*Bison bison*). **(a)** External examination of the body showing skin jaundice and presence of ticks (*Ixodes* spp.) on the inner side of the thigh (arrows), case no. 1; **(b)** Postmortem exam of abdominal cavity revealing petechial hemorrhages and echymosses on the digestive tract serosa, case no.1; **(c)** Cross section through the cerebellum showing multiple petechial hemorrhages (arrow), case no.2; **(d)** Multifocal petechial hemorrhages on the epicardium (arrow), case no. 2; **(e, f)** Representative photomicrographs of the histopathological changes observed in bisons infected with *Babesia* spp. (case no.3): **(e)** extensive renal tubular epithelium necrosis and ocasionally intratubular acidophilic proteinaceous material (arrow), and **(f)** coagulative necrosis of periacinar hepatocytes and hemorhages (arrow), HE stain, scale bar = 50 μm.

Out of 36 examined bison carcasses, in six cases, the macroscopic findings raised a suspicion of babesiosis: the post-mortem examination of these individuals revealed pale mucosae, mild to moderate jaundice, multifocal skin lesions associated with ticks (*Ixodes ricinus*) on the inner side of the thigh (Figure 2a), mammary gland, abdomen, ears and neck, petechial hemorrhages and echymosses on the digestive tract serosa (Figure 2b), cerebellum (Figure 2c), epicardium (Figure 2d) and pleura, splenic congestion, and severe dilation of the gallblader. Pulmonary congestion and edema, hydropericardium and reddish-brown urine were identified in only one case. Aditionally, the histological examination revealed extensive renal tubular epithelium necrosis (Figure 2e) and necrosis of periacinar hepatocytes (Figure 2f). Cytological evaluation of the spleen imprints was negative for the presence of intraerythrocytic piroplasms.

### 3.2. Molecular results

**Figure 3:**
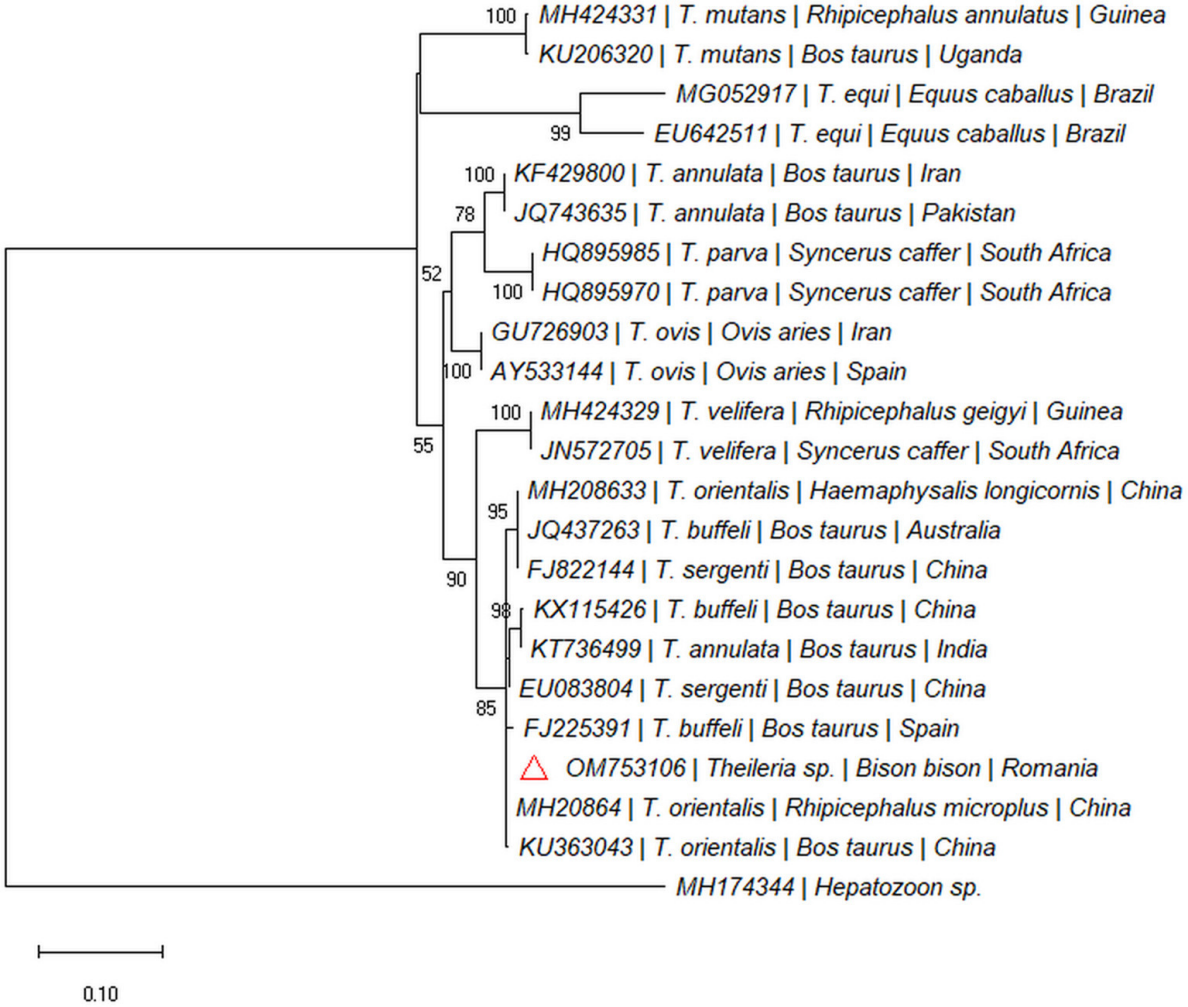
Pylogenetic tree constructed by maximum likelihood method on nucleotide sequences of *18S rRNA* gene of *Theileria* sp. *Hepatozoon* sp. was used as the outgroup.

One out of the six dead bisons (16.66%; spleen) was PCR-positive for the *18S rDNA* gene (Taga, Cluj County). The sequence was 100% similar with sequences of *Theileria* isolated from cattle: *T. orientalis* (e.g., MH208641), *Theileria annulata* (e.g., MF287924), *Theileria sergenti* (e.g., KX375823), and *Theileria buffeli* (e.g., KX965722) and clustered within the same clade (Figure 3). The sequence was submitted to the GenBank database under the accession numbers: OM753106.

**Figure 4:**
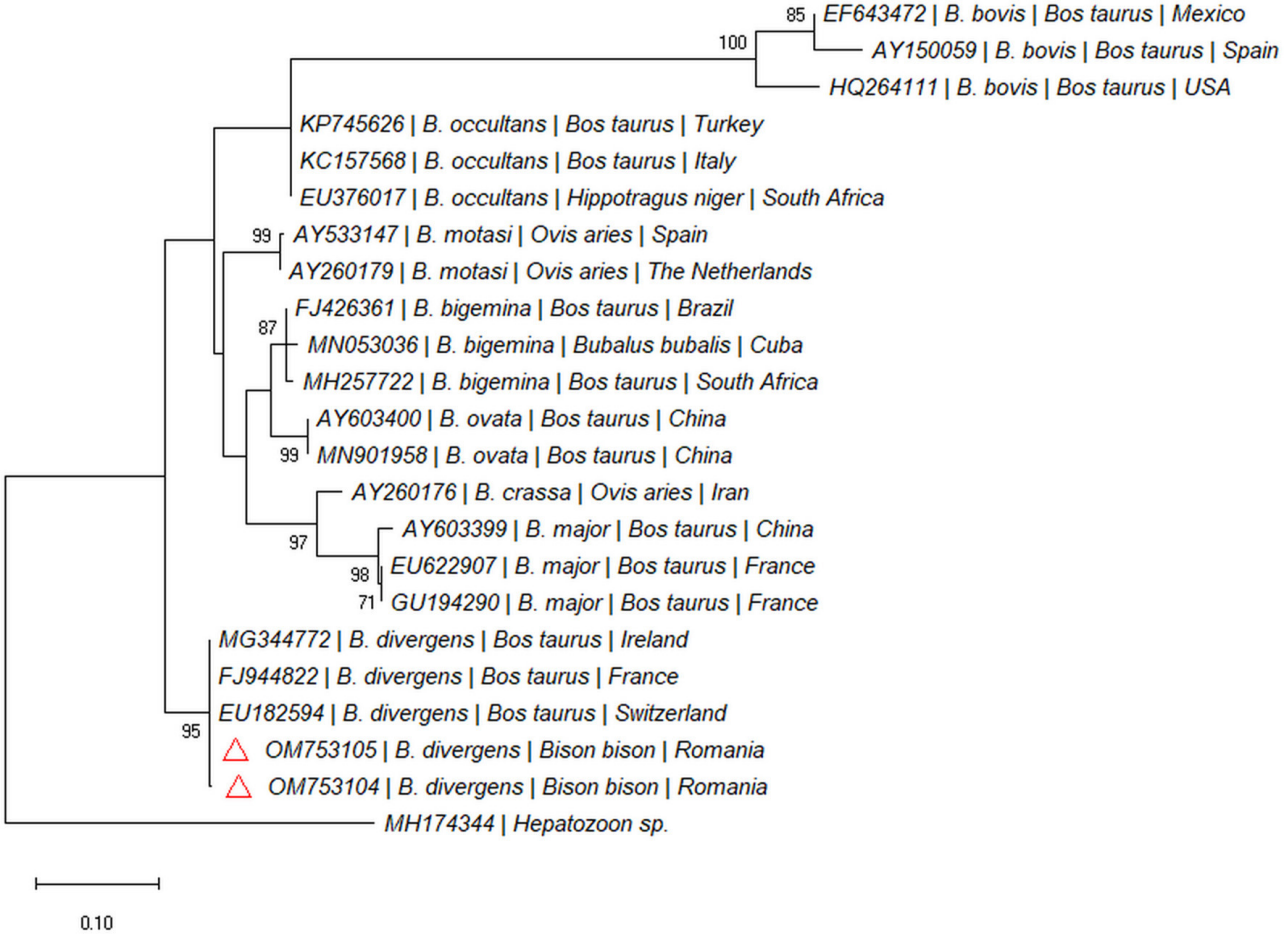
Pylogenetic tree constructed by maximum likelihood method on nucleotide sequences of *18S rRNA* gene of *Babesia* spp. *Hepatozoon* sp. was used as the outgroup.

Two out of 97 blood samples (2.06%) collected from live animals from Recea Cristur were positive for the *18S rDNA* gene of piroplasmids. We didn't detect piroplasmids in other two farms (Păticeu, Sărata). The BLAST analysis of the *18S rDNA* sequences for these positive samples showed a 99.79% to 100% similarity with several *B. divergens* isolates (e.g., MG344772, LC477141, and KY242398). The two sequences were highly similar to each other (99%), and formed a separate clade with *B. divergens* isolated from cattle in Europe (Figure 4). Both sequences were submitted to the GenBank database under the accession numbers: OM753104 and OM753105.

## 4. Discussion

As far as we are aware, in Europe there is no study focusing on the detection of vector-borne pathogens in *B. bison* and this is the first report of naturally occurring piroplasm infection in farmed American bisons from Europe. Babesiosis in animals and humans is caused by infection with tick-borne apicomplexan parasites of the genus *Babesia* (21). The present study reports the presence of *B. divergens* DNA in samples collected from live and dead bisons. The presence of *Babesia* was established by the association of the epidemiological, cytological and molecular findings. The animals were raised in farms for meat production and had access to pastures from which they could get the vector tick, *I. ricinus*. This tick species is known to be able to transmit, among others, several species of *Babesia* such as *B. divergens, Babesia venatorum*, and *Babesia microti* (both in humans and in animals) (22). The live animals from which the blood was collected were not searched for the presence of ticks. The ticks collected from dead animals were identified as *I. ricinus* in the Department of Parasitology and Parasitic Diseases, but no photo was taken then.

In Europe, different *Babesia* species can infect large ruminants: *B. divergens* (the main agent of babesiosis in cattle) (23), *Babesia capreoli* (free-ranging asymptomatic roe deer) (24), *B. venatorum* or *Babesia* sp. EU1 (25), *B. bigemina*, and *B. bovis* (in cattle) (26). *Babesia divergens* is also thought to be responsible for most European cases of human babesiosis (27). As this pathogen is transmitted by the ubiquitously distributed vector, *I. ricinus*, it can pose a threat of being transmitted to other animals and to humans. However, it is unknown if bisons can serve as reservoir hosts for this zoonotic haemoparasite.

*Babesia* in large ruminants can affect the health and economic productivity and management practices play an important role in limiting the spread of this parasite (28, 29). The overall impact of this disease on the productivity in the meat industry of bisons is still unknown, as the pathogenicity of piroplasms for bisons has been very poorly investigated. In USA, an experimental infection of *B. bigemina* was conducted in two splenectomized and one non-splenectomized bison calves. After the inoculation, all animals developed acute babesiosis with moderate to severe hemolysis, hemoglobinuria and hematuria (13). It was thus demonstrated that the American bison is susceptible to the infection with *B. bigemina*, showing once again that prevention and limiting the contact between wildlife and domestic animals is very important in order to avoid infections. Our study is the first report of *B. divergens* in American bisons.

Another protozooan parasite which can infect animals is *Theileria* sp. (30). The species that are known to infect large ruminants, especially cattle, are represented by *T. annulata* (tropical theileriosis) (31), *Theileria parva* (east coast fever) (32) and the *T. orientalis* group (*T. sergenti, T. buffeli, T. orientalis*) (33). *Theileria orientalis* group species are transmitted by ticks from the genus *Haemaphysalis* (34, 35). Different prevalences of infections with *Theileria* sp. were detected in cattle from Europe, ranging between 3.70 and 79.1% (36, 37), and the most common species identified was the group *T. sergenti/T. buffeli/T. orientalis*. The phylogenetic analysis of the *Theileria* sp. detected in our study showed that it clusters together with species from the *T. sergenti/T. buffeli/T. orientalis* group.

Our study showed the presence of piroplasmids in samples collected from *B. bison*, broadening the knowledge on the pathogens that can infect these animals. Further studies on prevalence and impact of babesiosis on farmed bison are recommended.

## Data availability statement

The datasets presented in this study can be found in online repositories. The names of the repository/repositories and accession number(s) can be found in the article/Supplementary material.

## Ethics statement

Ethical review and approval was not required for the animal study because all samples were collected during a necropsy (approved by the owner) or during sample collection for a national survey for disease. Written informed consent was obtained from the owners for the participation of their animals in this study.

## Author contributions

Conceived and designed the experiment and wrote and revised the paper: AC, MT, and AM. Performed the experiment: AC, TU, and MT. Laboratory work: AC and TU. Phylogenetic analysis: AI. All authors have read and approved the final manuscript.
