# Supplementary figures and images for: Piroplasms in farmed American bison, Bison bison from Romania
Source: Front Vet Sci. 2023 Mar 29;10:1158072. doi: 10.3389/fvets.2023.1158072 (PMC10090506; doi:10.3389/fvets.2023.1158072)

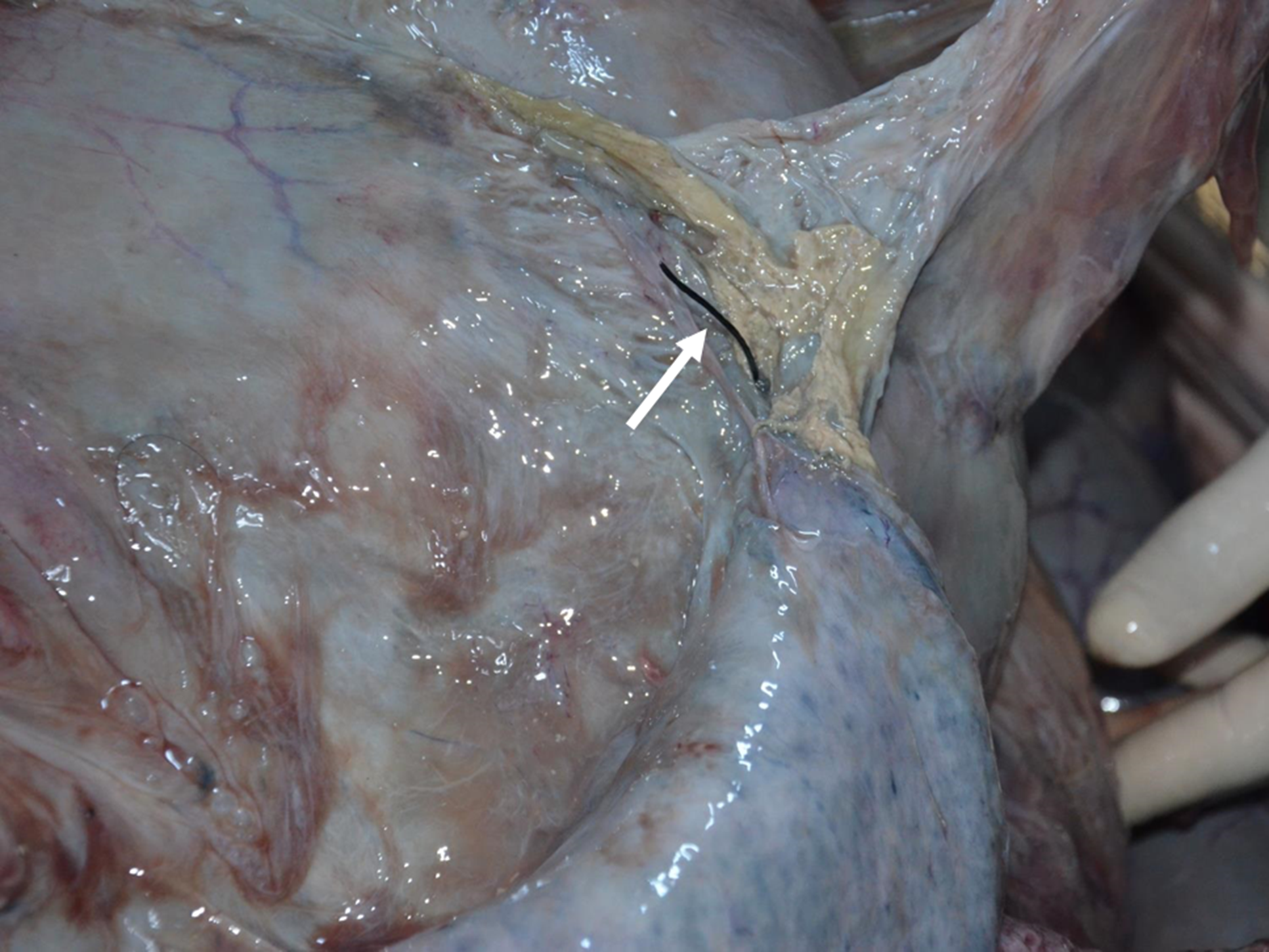

Supplement: Supplementary file 1 [file Image_1.TIFF]
